# Bibliographical Notices

**Published:** 1851-09

**Authors:** 


### BIBLIOGRAPHICAL NOTICES.

*Quarterly Summary of the Transactions of the College of Physicians of Philadelphia. From May 6th, to July 1st*, 1851, *inclusive.* Philadelphia: Lippincott, Grambo, & Co., 1851.

This number of the Transactions of the College of Physicians, is filled with excellent papers, and must, indeed, be gratifying to all who feel an interest in the maintenance of an independent organ by this venerable institution. We cannot too strongly commend so useful a publication to the notice of our readers; and we have no hesitation in saying, that it presents the greatest amount of valuable original matter, for the terms of subscription, with which we are acquainted.

The present number contains two highly interesting and elaborate communications from Dr. Pepper, of the Pennsylvania Hospital; one presenting a *case of Cancer of the Lungs and Mediastinum*, the other, on *Lemon-Juice as a remedy in Rheumatism;* a very remarkable *case of Hermaphroditism*, by Dr. Neill; a case of *Rupture of the Gall-Bladder*, by Dr. H. Hartshorne; a case of *Degeneration of the Liver, and Hypertrophy of the Gall-Bladder*, by Dr. Keller; and a case of *Death post partum*, by Dr. Beesley.

Dr. Pepper's paper on the use of Lemon-Juice in Rheumatism, gives us the details of fourteen cases, in which this remedy was employed in the Pennsylvania Hospital. The results which they present, are decidedly favorable to the treatment in question, in "acute uncomplicated rheumatism." And, even in the sub-acute or more chronic forms, it appears to have afforded marked relief in several instances; whilst under no circumstances, was it productive of an injurious result. Dr. Pepper, however, with characteristic caution, observes that

> "The number of cases just reported is entirely too limited to enable us to form any positive conclusion as to the curative powers of lemon-juice in this disease, particularly when we call to mind the numerous and sudden changes by which this affection is characterized, whether abandoned to itself or subjected to the various modes of treatment which have from time to time been recommended. It would have been both curious and instructive to have reported, in connection with the above cases, others of a similar character in which the lemon-juice had not been administered; but I have perhaps already wearied the patience of the college, and must hasten to bring these remarks to a close. I would simply state, however, that, during the month of March, of the current year, two cases of acute rheumatism were admitted into the hospital, both of which were treated chiefly by calomel and opium, with colchicum and magnesia; the first, though complicated with severe endocarditis, was fully convalescent by the end of the twelfth day; whilst in the second instance the relief was more prompt, the patient being nearly well by the expiration of one week. On looking over my notes on hospital cases for the last nine years, I also found many instances of acute inflammatory rheumatism recovering in the course of one or two weeks under this plan of treatment; on the other hand, it is true, there were cases of an apparently similar character reported, in which no such favorable results were obtained; and it would be important to ascertain, by further experience, whether, under such circumstances, the 'acid treatment' would be more efficacious. The very favorable reports from abroad certainly entitle this remedy to the attentive consideration of the profession; and, should it be found not inferior to the other plans of treatment, it would at least have the advantage of agreeable flavor, without, at the same time, impairing the general health, as sometimes happens from the use of more heroic remedies."

Dr. Pepper states that, in most of the instances in which the urine was examined, "it was found to be either neutral or much less acid than in health; whilst at the same time, it was unusually clear, and contained but a small amount of lithates; in no instance did it appear to be materially increased in quantity." Dr. Pepper's observations in this particular, coincide with those of Dr. Rees, the originator of the treatment.

Dr. Pepper offers no opinion as to the *modus operandi* of the lemon-juice; excepting that he attaches little influence to the citrate of potash which it contains. We find in a late number of the London Lancet, several cases of acute rheumatism reported, in which Dr. Rees employed citric acid, in place of lemon-juice, with a view of elucidating the question—what agency is attributable to the citrate of potash contained in the lemon-juice? One scruple of citric acid, dissolved in mint water, was administered every fourth hour, after a mercurial purge. He considered that the convalescence of the patients subjected to this treatment, was more tardy than with those treated by the lemon-juice. But this circumstance, he is inclined to think, may have depended on the peculiar state of the weather prevailing in London, this spring, which was very unfavorable to persons recovering from severe disease. In the practice of Dr. Keating, of the St. Joseph's Hospital, Philadelphia, pure citric acid exerted the same beneficial effects as lemon-juice, in cases of rheumatism.

Dr. B. H. Coates, who took part in the debate which was elicited by the reading of Dr. Pepper's paper at the College of Physicians, is of opinion that there is

"An error in the chemistry of the theory or explanation, by which good effects are demanded of the vegetable acids, in the treatment of rheumatism. The vegetable acids are not oxidating substances when introduced into the animal economy; they are de-oxidating or dis-oxygenizing substances. They carry more oxygen out of the animal body than they bring into it; and this loss is supplied at the expense of the blood. It is granted that these substances are never found among the excreta; the matters of which they are composed are, when discharged, combined in the form of carbonic acid and water, either separate, or having the carbonic acid combined with alkalies or lime, found in the composition of the fluids. When converted into carbonic acid and water, the vegetable acids always absorb oxygen, necessary for the composition of the two resulting products.

This corresponds with the chemical theory that gouty matter is in want of further oxidation preparatory to its discharge by the kidneys; and with the usual experience, that vegetable acids render gout worse, or produce it. The analogy of gout to rheumatism, on which the speaker did not then insist,is adverse to the hope of benefit from the use of vegetable acids in rheumatism."

Dr. Coates considers any good effects that may result from the use of lemon-juice in acute rheumatism, to depend entirely on the *sedative* and *refrigerant* action of the remedy, and to be effected in spite of its deoxidating action. The modus medendi, he deems the same, as with "the moderate and rational use of bleeding and of the nitrate of potassa." And these views he finds agree with the observations of Dr. Pepper, who derived little advantage from the lemon-juice in *chronic*, but only generally or frequently so in *acute* rheumatism. "Acute rheumatism is rheumatism accompanied by fever; and the use of sub-acid drinks, for the sake of their sedative effects in fever, is familiar, and has descended to us from former ages. * * * * * * * In this, way Dr. Coates sees no impediment to lemon-juice being serviceable in acute rheumatism, doing some harm, but more good."

---

*An Address on Medical Jurisprudence, delivered before the Fellows of the Massachusetts Medical Society, at the Annual Meeting, May* 28, 1851. By David Humphreys Storer, M. D. Boston, 1851.

This address is a well-timed and pointed appeal in behalf of the claims of *Medical Jurisprudence* to an increased share of professional attention. Dr. Storer justly observes, that, while

"With all the other departments of the profession, the young man, presenting himself for his final examination, may be sufficiently well acquainted, to satisfy the Faculty before whom he appears, that he is competent to commence the responsibilities of his career, upon the subject to which I have referred, in some of our schools he is *not examined:* not a question is asked him. He may have listened to a few general lectures during his pupilage; but he has pursued no methodical course of instruction,—he has studied no accurate, reliable author,—he has had no recitations upon the subject; without which, in this, as in all other branches, lectures are of comparatively very little value. And it is only when he is summoned to give his testimony in a Court of Justice, that the young physician finds himself forming his opinion, and searching for authorities to support it; or perhaps, I might with more propriety say, collecting published facts upon which he may found an opinion."

It is, indeed, surprising that the important subject of Medical Jurisprudence should be so completely over-looked in some of our Medical Schools. And we cannot but welcome with satisfaction this appropriate discussion of a very desirable reform.

The following forcible remarks on the toleration of irregular practitioners, from Dr. Storer's address, we commend to general perusal. They are no less applicable to our own meridian than to that of Massachusetts:

"Much yet remains to be done to perfect our system of medical police. In the year 1781, several of the most distinguished physicians of the State associated themselves together, and obtained an act of incorporation from the Legislature, under the name of the 'Massachusetts Medical Society.' By their charter, they are expected 'from time to time to prescribe such a course of medical and surgical instruction, and such qualifications as they shall judge requisite for candidates for the practice of physic and surgery, and shall cause the same to be annually published.' This course is pursued; and well-educated young men yearly present themselves to the proper officers to be examined, and, proving themselves to be competent, are allowed to become members of the Society. But we look in vain in the chapter 'concerning the practice of physic and surgery,' in the State's laws, for a restraint upon irregular practitioners,—for a prohibition, that none save well-educated men, and such as have shown their capability by undergoing a thorough examination at the proper tribunal, shall be allowed to act the part of a physician or surgeon. And the young physician, a member of the Massachusetts Medical Society, who has been able by the most strenuous efforts,—by great self-denial,—oftentimes by embarrassing himself for years, in a pecuniary point of view, to reach the goal for which he has so long and so ardently striven, finds, upon entering the threshold of his profession, that he is surrounded by ignorant, uneducated, unprincipled men, who have no hesitation in publicly proclaiming that they can cure all diseases; and that, too, without resorting to any of those remedies against which they know the people have an insurmountable objection,—men who deluge the community with their hand-bills and certificates of the most remarkable success, prepared for the occasion, or testified to by bribed or irresponsible persons.

In no respect is our medical police more inefficient than in this. The evil I speak of has become a public nuisance, and as such it should be treated. A great portion of every community are exceedingly credulous,—believing most fully whatever may be stated, which, to an enlightened mind, savors of impossibility. The more ridiculous and improbable the accounts, the more readily do they attract attention; and the greater the audacity of the narrator, the more certain, for a period, is he of succeeding.

I have known a delicate female, wasted with phthisis, and requiring all the sympathy and attention of her most devoted friends, persuaded to place herself under the care of one of those wretches who blasphemously warrants a cure, and subjected to the most active treatment that could be devised. A few days only were required to free her of her misery.

I have seen a strong day-laborer, treated for inflammation of the bowels with the most stimulating drinks, crying in his agony for cold water, and supplied with potations of rum and cayenne, and compelled, in his intervals of repose from the most acute suffering, constantly to repeat the dose.

A few years since, a villain, who was said to have graduated from a Southern State prison, practiced in our metropolis with immense success. Mercury and venesection, in his hands, controlled all diseases. The former in spoonful doses, and the latter to the utmost limit of the patient's strength, were employed indiscriminately. To use his own words, openly expressed and openly boasted of, "he had drawn barrels of blood.' Gross as were his proceedings, numerous as were the victims of his malpractice, there he remained outraging the community, until the relatives of a patient he had imposed upon and ruined made a public exposition of the case.

But why should I adduce individual instances to prove my position, when, with others equally striking, most of you are undoubtedly familiar? Besides, the physician, as such merely, can do but little in this hoped-for reform. However anxious he may be to do his duty as a good citizen, to exhibit the villainy which exists on the one hand, and the unavoidable misery consequent upon it on the other, but few can appreciate his motives, or will give him credit for disinterestedness; and he is literally compelled not only to see the grossest impositions inflicted upon his fellow-men, but to feel also, that any interference on his part is the surest means of increasing them. It should be the duty, therefore, of those whose education and condition in life enable them to observe and comprehend the existing evil, to endeavor to remedy it. The better part of the community should act in unison upon this subject, and then the object could be accomplished."

---

*Lectures to a Candid Inquirer, on Animal Magnetism.* By WILLIAM GREGORY, M. D., F. R. S. E., Professor of Chemistry in the University of Edinburgh. Philadelphia: Blanchard & Lea. 1851.

This book is a lamentable specimen of folly and credulity, from a quarter whence better things were to have been expected. It is, indeed, humiliating to find a person who occupies the professional and scientific position of Dr. Gregory, assuming the championship of charlatanism, and lending the sanction of the venerable university with which he is connected, to what all right-minded members of his profession view with unmingled contempt and scorn. We really cannot understand what the professional feeling of the ancient Delphos of medicine can be, when the Professor of Chemistry in the University takes up the mantle of Mesmer, and the President of the College of Physicians* openly avows himself a disciple of homœopathy.

Dr. Gregory's book is, we are bound to say, apparently written in good faith and candor; and we must give him credit for being a true believer. He betrays, however, a degree of credulity, which in a man of his pursuits and presumed train of thought, is, indeed, astounding; not only admitting and defending every variety of the mesmeric humbug—clairvoyance, phreno-magnetism, and all—but gravely narrating and adopting the most monstrous stories, a belief in which overthrows every received opinion, and implies a rejection of all knowledge, natural and revealed.

It would be absurd to deny, that the influence of strong-minded upon weak-minded persons is capable of producing, under certain circumstances, a train of irregular nervous phenomena, which we cannot with entire satisfaction explain. These phenomena have been seized upon and made the basis of the most reckless pretensions and falsehoods, which have been again and again exposed, and as often revived. It is useless to continue the task of unmasking these delusions, for argument and reason have no weight with the excitable and weak-minded persons who entertain them.

We give some samples of Dr. Gregory's twaddle and silly stories. He proposes the name of *odyle* for the principle heretofore termed animal magnetism, which he assumes to be "universally diffused, like heat, light and electricity." Like them, "it tends to a state of equilibrium, and its external manifestations seem to depend chiefly on the disturbance of this state of equilibrium." While ranking, however, *odyle* with the known imponderable agents, he has the grace to admit "that we know comparatively little of the laws by which it is regulated," particularly, as "hitherto observers have not been able to examine the most important facts by the aid of their own senses, but have had to trust to the sensations of others." It is also admitted, that "we do not yet possess any means of collecting, accu-

* Dr. Henderson.

mulating and concentrating the odylic force, as we can do in the case of magnetism and electricity." "Nor have we yet obtained a convenient and accurate means of measuring the quantity or intensity of odylic force."

As an apt conclusion of this rhodomontade, we select one from numerous cases illustrative of "sympathetic clairvoyance," which Dr. Gregory relates, as entitled to implicit credence. "Sympathetic clairvoyance" is the power of reading the thoughts or perceiving the state of health of persons *en rapport* with the sleeper. And the relation in question is established either directly by contact, or indirectly by means of their writing or a lock of their hair! By the aid of handwriting, one of Dr. Gregory's *clairvoyantes* is made to detail the thoughts and actions of persons of various times and countries, including Mary, Queen of Scots, and other notabilities. The following pretended account of Sir John Franklin is, we must say, the most impudent specimen of humbug we have ever met with. And we believe that information received since the publication of Dr. Gregory's book, leaves little doubt that, far from Sir J. F.'s having been "met and relieved" last winter, his ill-fated expedition had long previously been destroyed.

"It is pretty generally known, that this clairvoyante was tried with the writing of Sir John Franklin, and a part of what she said has appeared in the newspapers. I had the opportunity of becoming acquainted with what she did really say, and, although of course the greater part of it cannot be verified until the return of Sir John, yet I am bound here to testify, although she has probably mixed up and confused many things, which we have not the means of distinguishing, that E. has said nothing concerning him which may not prove correct. It appears that some clairvoyants, of whom I know nothing, went so far as to predict the return of Sir John during last autumn. If such predictions were made, by genuine and honest clairvoyants, I conjecture that they have been of that class, who are strongly affected by sympathy with the feelings and wishes of those who consult them, which feelings and wishes they, as it were, reflect. But this is not the case with E. She has made no prediction in the matter, but has simply, at various times, with the aid of Sir John's handwriting, gone, in her phrase, to see him. She was not told, and does not, I believe, even yet know, whose writing it was; but she found the writer in one of two ships, fixed in ice, and surrounded with walls of snow. These ships she first saw in the winter of 1849--50, I believe; I saw several of Dr. Haddock's letters about it in February and March, 1850. Since E. had been right in so many cases at a distance, it was probable that she was also right in this one. She described the dress, mode of life, food, &c. of the crews. She saw and described Sir John, and said that he still hoped to get out, but was much surprised that no vessels had come to assist him. She frequently spoke of his occupations, and when asked the time of day, found it either by looking at a timepiece in the cabin, or by consulting Sir John's watch. During the winter and spring of 1849—50, and part of the summer of 1850, she uniformly indicated the same difference of time, which I cannot at present give precisely, but which was nearly seven hours. At whatever hour she was magnetised and sent there, she always made the same difference. Nay more, when the time there was nine or ten A. M. (four or five P. M. at Bolton) she would say that such was the hour, but that it was still dark, and lights were burning in the early part of summer. Now it is quite absurd to suppose that this totally uneducated girl has any notion of the relation of longitude to time, or of the difference between an arctic day and one in our latitude. E. also being shown the handwriting of several of the officers of the expedition, found and described them. One was dead (shelled, as she said,) when she was asked. Another, at a later period, was dangerously frostbitten, but recovered. She said, that in one of the ships the provisions were exhausted, but that the other contained provisions. She described the fish, seals, and other animals hunted and killed for food and oil by the crews. Of, or rather to, one officer she said that he was the doctor, although not dressed like a doctor, but like the rest, in skins; that he was a first-rate shot, and was fond of killing animals to preserve them. (This is really the case with Mr. Goodsir, whose writing she was then examining.) She added a multitude of curious details, for which I have no space, and they will no doubt be published by Dr. Haddock. But I may mention, that on a Sunday afternoon in February 1850, she said it was about 10 A. M. there, and described the captain (Sir John) as reading prayers to the crew, who knelt in a circle, with their faces upwards, looking to him, and appearing very sorrowful. She even named the chapter of St. Mark's gospel which he read on that occasion. She also spoke, on one occasion, of Sir John as dejected, which he was not before, and said that the men tried to cheer him up. She further spoke of their burning coarse oil and fish refuse for warmth, and drinking a finer oil for the same purpose. All this time she continued to give the same difference of time, from which the longitude might be calculated. This time, seven hours, or nearly, from Bolton, gives a west longitude of about 100° to 115°, which corresponds very well with the probable position of Sir John. But at a later period, all of a sudden she gave a difference of time of somewhere between six and seven hours, indicating that the ships had moved eastward. She was not, after this, quite so uniform in the difference of time as before, and seemed not to see it so clearly; but she persisted that they had moved homeward, and if we take about 6½ hours as the later difference, this would indicate a longitude of about 97° 30′ W. After this change, she also said that Sir John had been met and relieved, and has always since then seen three ships, which, for a long time past, are said by her to be frozen np together. The last observation of which I have heard, 17th February 1851, gave a longitude of 101° 45′ W. At the same time, from Captain Austin's writing, which has also been frequently tried, she gave for him the longitude of 95° 45′ W. She does not know whose ship it is, that according to her, has met with Franklin, but she still speaks of three ships together. I should add, that when E. has been sent there at such an hour and season that it was night in those latitudes, she has, quite spontaneously, described the aurora borealis, which she once saw, as an arch, rising as if from the ground at one end, and descending to it again at the other. From this arch, colored streamers rose upwards, and some of these curved backwards. She was much surprised and delighted with it, and asked if that was the country the rainbow came from. She had never been told anything whatever about the aurora, and knows nothing of it."

---

*Elements of General and Pathological Anatomy, presenting a view of the present state of knowledge in these branches of science.* By David Craigie, M. D., F. R. S. E. Fellow of the Royal College of Physicians, of Edinburgh, &c. &c., *Second edition, enlarged, revised and improved.* Philadelphia, Lindsay and Blakiston, 1851.

The new edition of Dr. Craigie's admirable work comprises all that was contained in the previous issue, with such rectifications as the advances of the science rendered necessary, with the addition of two new books, one on the structure and morbid state of the glands, and the other on the structure and morbid states of the lungs and heart.

To those who are familiar with the previous edition, we need say nothing in commendation, its merits being fully recognised. In the work before us, the same arrangement, based upon the distinctions of the component tissues of the animal body as derived from the similitude and difference of their anatomical character, and which was so advantageously used in the first edition, is maintained. The object of the author to communicate precise and useful information in a perspicuous and methodical manner has thus been accomplished, and the lover of this department of science, will find prepared to his hand, an accurate representation of all that is known in it.

*Of Happiness in its relations to Work and Knowledge. An Introductory Lecture delivered before the Members of the Chichester Literary Society and Mechanics' Institute, October 25th, 1850, and published by their request.* By John Forbes, M. D., F. R. S., Physician to her Majesty's Household. Philadelphia: H. Perkins. 1851.

Of every creature under heaven, the unceasing demand has always been "who will show us any good?" Every avenue to happiness is therefore thronged with earnest seekers for that "great good," and every prophet who professes to have seen it, and to be able to indicate to others the course in which it may be pursued with reasonable hope of success, is sure of an anxious crowd of followers. Unfortunately, all are too prone to think it lies in some other direction than that in which their own path leads them, and are, therefore, ready to bolt over every hedge and ditch that bounds their regular career, in hope of avoiding rough places they see in prospect, or the recurrence of difficulties which they have already surmounted at an expense of toil they are reluctant to feel liable again to exert. More attractive works than "searches after happiness" could not present themselves. Yet few who are longing for it will think that its "Relations to Work and Knowledge" are so intimate as are represented in the Essay on this subject, from the pen of Dr. Forbes, whose title stands at the head of our notice. The inimitable Flaccus, when looking back from the highest point of literary honor and elegant ease, upon the scene of earlier joys on the paternal farm, could assert of those who cultivated the fertile vales of Italy, "Nimis beati si sua bona norint." And the poet philosopher, from the chair of a Scotch University, could announce a truth which first fell upon our ears, in the short intervals between toil, afforded to a half-grown boy on a farm cultivated by the personal exertion of the family:

> "From labour, health, from health, contentment springs;
> Contentment opes the source of every joy."

But, unfortunately, these and the numberless other testimonies to the effect of labor in promoting that happiness we all desire, come from sources which are liable to question; as the witnesses do not give that unerring test of confidence in the truth of their own teachings, which practice furnishes, but unfortunately, must be comprised in that large class of teachers, who "point the way they never mean to go."

Yet in nothing do observation and practical philosophical induction more accurately coincide in their teaching. The energetic activity of early life, whether of the lower animal, or that connecting link of the animal and spiritual existences, man, displays itself in exertion, for which no motive can be conceived, except the mere gratification which arises from putting forth of power. Who does not accord to this period the epithet by which it is universally recognized, of "Happy Childhood"? The very memory of it is "redolent of joy and youth," and casts its reflected light with softened rays, over the else dim evening of mortal existence, and converts the very vapors of the approaching night into trophies of beauty as they float around it. But who would, for a moment, dream of the happiness of children separate from exertion? Every power, mental and physical, is brought into active play; perceptive, imaginative, reflective powers, are all busy. Nor is that combining power, by which all are brought to bear upon some effectual result, held in abeyance for future use. How many abortive schemes are brought to the test of actual experiment! How quickly is each unsuccessful effort followed by another and another still!

In manhood, too, we ever find that the sanguine temperament which retains most resemblance to youth, is most productive of available enjoyments to its possessor. But it is not from observation only that we derive the knowledge of this truth. It must be so from the necessity of the case. The fitness of things requires it. Toil, mental and physical, is required to develop the resources which are treasured up in the bosom of created matter, for the benefit of animated nature; and, whether it be the uncertain soaring of the butterfly on gaudy wing, the direct flight of the bee to the treasured nectar, or the combined mental and corporeal effort of man to bind the lightning to his service, and bring the winds into subjection to his power, active effort, positive toil, are everywhere made necessary to securing that which is appropriate to the support of each grade in animal existence. Even rest can have no sweetness unless preceded by the labor which requires its refreshment.

Happiness is not found in any extent of acquisition, and this is as true of mental as of physical treasures. The philosopher who should attempt to retire on a competency of knowledge, would present a spectacle quite as pitiable as that afforded in the pages of Dr. Forbes, and too often witnessed by wary observers, of the retired mechanic, merchant or professional man. It is only as knowledge attained affords a new point on which to rest the ever active mental lever, and move fresh obstacles to onward progress, that it confers happiness; and the moment we reached the point beyond which nothing more was to be known, we should be as wretched as

> "He who his tiara tore,
> And wept for want of other worlds to conquer."

Dr. Forbes, lecturing before a body which prohibits the introduction of religion or politics, is careful to avoid treading on ground which might be considered questionable, from its relation to either subject. The only point in which we are disposed to hesitate in adopting his views, is to be found where he considers the relation between labor and happiness so intimate, that even vice may not separate them. At least, such is the construction of which we think his language is susceptible; though writing, as we do, without access to the pamphlet, merely from the impression made by a careful perusal, we are unable to quote it accurately. There certainly is a temporary gratification of some individual passion or desire which may be said to afford pleasure. But we question the propriety of that definition of happiness which would describe it as merely prolonged pleasure. It is one of those things which it is more easy to comprehend than to explain. It is so intimately allied to the sensations and emotions, that it is difficult to bring it within the scope of verbal definition. We should be inclined to assert, that, while pleasure results from the healthful exercise of each of the varied organs of the body and faculties of the mind, happiness is the aggregate result of the combined and harmonious action of the whole. Its degree varies in proportion to the harmony which is maintained, and is higher and more refined just in the degree in which the nobler elements of our compound being are allowed their proper preponderance. While, then, we recognize a Creator, by whose wisdom all things are arranged as

"Parts of one stupendous whole,"

we must assume that *he* is likely to have the largest amount of happiness, who best fulfils the duties of the position in the world to which he has been assigned; and with the retrospective poet, who sings of the happiness of earth from the seats above, admit that "Where duty went, she went." The heathen poet gives to "Mens sibi conscia recti," the highest point in the scale of enjoyment.

When, then, we regard man as a compound creature, and refer to his animal, intellectual, and moral endowments, we must take the ground that each of this series of faculties must be brought into actual exercise, to open every avenue of enjoyment which is his privilege; and, also, that the highest enjoyment must be commingled with the nobler faculties. The man who sinks his intellectual and spiritual nature, may attain to animal enjoyment. He that cultivates his intellectual power, will add to this the higher gratification of intellectual delight. But he who, not unmindful of these, roams abroad through the fields of regulated animal enjoyment, and soars, at will, into the higher regions of intellectual space, realizes that there is beyond, an unlimited expanse, filled with suns—of some of which the light twinkles in the blue serene, while of others, no ray has yet travelled down to our dull planet,—and has a source of enjoyment opened up to his contemplation, which leaves far in the dim distance of earthly shade, the highest point to which the devotee of animal or intellectual pleasure has ever attained. *Perfect happiness is to be found in the full play of* ALL *the powers of man.*

Although not strictly within the limits of criticism in a Medical Journal, we have been induced to bring the essay whose title heads this article before our readers, and to commend it to them as a chaste and classical production of one whose name has long been associated with the literature of our profession. And we offer our thanks to Mr. Perkins for placing it within the reach of all. While the American edition of this little work, is worthy of much praise for its neatness, we must be allowed to enter our solemn protest against the curtailment of the original,—or rather against the reason given for the omission of a part. It is a libel on the reading community of America, to infer that it will pardon the leaving out of the "Latin." Print it, and let him who can, read, and let it stand as an incentive to him who can not, to put forth his "Labor" to acquire that "Knowledge," and in doing so he will find "Happiness." **

---

*The Microscopist; or a complete Manual on the use of the Microscope: for Physicians, Students and all lovers of Natural Science. With Illustrations.* By JOSEPH H. WYTHES, M. D. Philadelphia: Lindsay & Blakiston. 1851.

The above is the title of a neat duodecimo volume of one hundred and eighty-two pages, intended to fill a deficiency long felt by those desirous of pursuing microscopy, yet who were unable to obtain access to the more elaborate treatises on the subject. It contains fourteen closely printed chapters, and is handsomely illustrated by wood engravings. A brief enumeration of the contents will show the amount of material contained within its limits. Chapt. I. The history and importance of microscopic investigation; Chapt. II. The microscope; Chapt. III. Adjuncts to the microscope; Chapt. IV. How to use the microscope; Chapt. V. On mounting and preserving objects for examination; Chapt. VI. On procuring objects for the microscope; Chapt. VII. Test objects; Chapt. VIII. On dissecting objects for the microscope; Chapt. IX. The cell doctrine of Physiology; Chapt. X. Examination of morbid structures, &c.; Chapt. XI. On minute injections; Chapt. XII. Examination of urinary deposits; Chapt. XIII. On polarized light; Chapt. XIV. Miscellaneous hints to microscopists. It will thus be seen that the student and beginner have presented to them in a cheap form an amount of matter that, we are sure, will whet their appetites for the more elaborate treatises on the various subjects; and if they be true lovers of the science, they will not stop until they have mastered all.

In glancing over the chapter on minute injections, our attention was arrested by one or two matters of detail, wherein slight inaccuracies have occurred. For instance, on page 139, the author, in describing the "chemical process" of making minute injections by throwing into the vessels first the solution of bichromate of potass, and then following it by an aqueous solution of acetate of lead, describes it as due to M. Doyere, and first published in the *Comptes Rendus* for 1841. In the number of the American Journal of Med. Sciences for Aug., 1833, p. 353, the author will find a paper by Prof. Horner, in which he describes a mode of making fine injections by the use of solutions of sulphate of iron and prussiate of potash, as well as that by chromate of potash and acetate of lead. The suggestion of their use is there credited to Dr. Goddard of this city. Its assumption by M. Doyere is a fair illustration of the story of Columbus and the egg! Again, on page 140, he quotes Dr. Goadby's paper upon the same process with the addition of gelatin, which was copied into this journal, with the corrections of Dr. G., in March, 1850, wherein Dr. G. "suggests the substitution of the *nitrate* for the *acetate* of lead, as we would then have, in the liberated nitrate of potash, a valuable auxiliary in the process of preservation." If we are not very greatly mistaken, Dr. Goadby's own experience now will controvert this, as the nitrate of potassa dissolves the chromate rapidly, and in a few hours destroys the injection.

One other point, also, and we have concluded all in our notice that is not praise. On page 141 it is said that another plan has been *suggested* (the italics are our own) by Dr. Goddard, viz., that of suspending finely levigated coloring matter in sulphuric ether and injecting the fluid into the vessels. The above-mentioned plan has been most successfully *used* by Dr. G., as well as by many others in this and other cities, and is therefore a *settled fact*, and not a mere suggestion. The author's failure in its use, we suspect, arose from his not using ether enough, under which circumstances it will sometimes clog up the vessel from the too early deposition of the coloring matter. The original paper, with full directions, will be found in the Examiner for Dec., 1849.

With these one or two exceptions, we have derived sincere pleasure from the perusal of this little work, and we earnestly commend it to all who are desirous of obtaining information in this most interesting and useful department of medical science.

*Minutes of the Proceedings of the Medical Society of the State of Georgia, at its second Annual Meeting, held at Atalanta, on the 9th April,* 1851. Savannah, 1851. pp. 30.

The late meeting of the Medical Society of Georgia appears, from the "Minutes of the Proceedings," to have been well attended, and to have passed off with much harmony and spirit. Dr. Arnold, of Savannah, was chosen President, with Drs. Means and Campbell, as Vice Presidents; Drs. Quintard and Nottingham, Secretaries, and Dr. Alexander, Treasurer. Standing Committees on *Medicine*, *Surgery*, *Obstetrics*, *Hygiene*, and *Indigenous Botany*, were appointed, with Drs. Bulloch, Campbell, Miller, Stewardson, and Quintard, respectively, as Chairmen. Appended to the transactions of the Society, are an abstract of a report on "the character of Education necessary for the Physician," by Dr. Ford, and an address by the President, Dr. Arnold.

Dr. Ford's recommendations on the subject of the preliminary education which should be required of Medical Students are elevated and judicious. He dwells strongly, however, on the necessity of legislative interference to attain any desirable reform in this particular.

"Whilst the Committee entertain the opinion that of all the subjects which have occupied the attention of the National Association, there is none of equal importance to this,—they are utterly at a loss to make any distinct propositions on the subject. The hopelessness of any efficient measures to accomplish this end, within the jurisdiction of our Society, arises from the opinion that the root of the evil lies far beyond our reach—it is to be found in the absence of all legislative protection of the profession, and the positive recognition by statute of every kind of practitioners of Medicine. With this legislative invitation to ignorance, incapacity and worthlessness, to come in and swell the ranks of our profession, and with that easy courtesy of society, through which any may attain the highest title of the profession, any attempt to demand a good general education as a qualification of pupilage, either in private offices or in the Medical Colleges of the State, would but increase the evil."

These views are certainly sound. *Reform* is an idle word, until the regularly educated practitioner is sustained by the strong arm of the law. And we believe that united and determined effort on the part of the profession, can obtain from our State

Legislatures the legal protection which is necessary to the improvement of our body. The concert of purpose and action on which the accomplishment of this result depends, will, we trust, grow out of the State organizations which have been so auspiciously brought into existence by the National Medical Association.

Dr. Arnold's address on "the reciprocal duty of Physicians and of the Public towards each other," is a manly and sensible effort, which cannot fail to have the best effect both in and out of the profession. He takes no half-way ground in his requirements from aspirants to the study of medicine, but boldly proclaims the opinion, that a thoroughly liberal preliminary education is the only basis upon which proper medical attainments can be built. "At the very least, in addition to a thorough English education, he would require from a young man undertaking the study of medicine, that he should be well versed in the Greek and Latin languages." In commenting on the duties of the Public towards Physicians, Dr. Arnold alludes, with admirable force and temper, to the many difficulties which popular prejudice throws in the way of professional improvement and advancement—the impediments to the study of practical anatomy—the opposition to the registration of births, marriages and deaths—the legislative privileges granted to every description of quacks, &c. We should be pleased to have room for his remarks on this point entire, and we trust that his address will have a general as well as professional circulation.

